# A gap between reasons for skilled use of BCI speech devices and reasons for utterances, with implications for speech ownership

**DOI:** 10.3389/fnhum.2023.1248806

**Published:** 2023-10-17

**Authors:** Stephen Rainey

**Affiliations:** Ethics and Philosophy of Technology, Delft University of Technology, Delft, Netherlands

**Keywords:** BCI, speech, pragmatics, ownership, reasons

## Abstract

The skilled use of a speech BCI device will draw upon practical experience gained through the use of that very device. The reasons a user may have for using a device in a particular way, reflecting that skill gained via familiarity with the device, may differ significantly from the reasons that a speaker might have for their utterances. The potential divergence between reasons constituting skilled use and BCI-mediated speech output may serve to make clear an instrumental relationship between speaker and BCI speech device. This will affect the way in which the device and the speech it produces for the user can be thought of as being “reasons responsive”, hence the way in which the user can be said to be in control of their device. Ultimately, this divergence will come down to how ownership of produced speech can be considered. The upshot will be that skillful use of a synthetic speech device might include practices that diverge from standard speech in significant ways. This might further indicate that synthetic speech devices ought to be considered as different from, not continuous with, standard speech.

## 1. Introduction

Brain Computer Interfaces (BCIs) designed to provide speech will have different technological and software features that will cause some limitations. For instance, every system will have a general error rate which may be higher or lower depending on the system's properties and the user's ability to operate it. Some systems may be better at classifying some inputs than others (e.g., vowels rather than consonants), and so require more from a language model to fill in the gaps. Or again, systems may include user “go” or “veto” commands to boost user control over output that introduce delays between user input and output. Importantly, the use of any such device by a user will be an acquired skill gained over time (McFarland and Wolpaw, 2018). These examples, and other such overall system limits, could have significant impacts on how much ownership a user may be able to claim over their BCI mediated speech outputs.

“Ownership” in this context is derived, though not lifted directly without modification, from Fischer and Ravizza's (1998) account of responsibility. In Fischer and Ravizza, ownership over action is discussed in terms of how closely an action can be related to the reasons an actor has for performing that action. A feature of being a morally responsible agent in general is “ownership” of the mechanisms that produce action. Owning these mechanisms includes building up dispositional beliefs about their reliability with respect to reasons, i.e., that one comes to expect one's reasons to reliably bring about predictable outcomes by means of the mechanism in question (Fischer and Ravizza, 1998, p. 218). This points to a historical dimension of action-producing mechanisms—that the actor has a proven ability to use them. This kind of ownership might be produced over time through the acquaintance with one's own body, for example, and confidence in one's dexterity. We might imagine a case in which a surgeon takes on difficult surgeries in the faith that she is well skilled and can produce good results. Here, she takes responsibility for her surgical outcomes, and can be held responsible. In Fischer and Ravizza's terms, she has “guidance control” in that she is skilled in the techniques and practices of surgery and knows what to do in surgical contexts. Less prominently featured is another dimension of control—regulative control—which would explain moral responsibility in terms of alternative possibilities. On this account, responsibility attaches to an actor's having brought about one alternative over another by acting as they did. It is enough to hold the surgeon responsible, on the account developed in Fischer and Ravizza, that she acquires her reasons for action, relates to them appropriately, and enacts them reliably to bring about expected effects. Hence, this is ultimately a compatibilist position, based in historical conditions enjoyed by an agent, that need not engage with the idea of the future and the past being in any way causally unrelated. Regulative control would mean control in a sense related to open possibilities in a metaphysically libertarian sense. As has been argued elsewhere (Rainey, 2022), in the case of synthetic speech action, however, it is not as clear as this.

Ownership over synthetic speech is at least a two-dimensional matter—first, there is the dimension of speech production and second, the speech action. Ownership over the former means much the same as with the surgeon—reliable engagement with whatever mechanism of BCI is gained and confidently exercised by the user. Ownership over speech action, however, takes on a different dimension owing to the ways in which the actions performed with utterances are open to interpretation by audiences, or the ways in which they can be ambiguous. Ownership over synthetic speech has an extra epistemic dimension in this sense, such that an utterance may be produced exactly as intended and may be entirely semantically accurate, yet be interpreted in a way the speaker would not endorse (Maslen and Rainey, 2020). Endorsement is connected to ownership through permitting a check that the speaker indeed would stand by their utterance, including where it has been interpreted in a particular way and reasserted to them.

It has been argued elsewhere, too (Rainey, 2022), that neuroprosthetic speech in particular requires that greater attention be paid to regulative control than guidance control, precisely to respond to the epistemic variety latent in speech production:

“…the neuroprosthetic speech case is one in which the proper realization of unimpaired volition is what is at stake—the responsiveness of neuroprosthesis users' speech to their own reasons. This requires an augmented role for regulative control because it is this kind of control that can identify an actual causal sequence.

Rather than helping oneself to metaphysical alternatives, in the sense of an opposition between free will and determinism, this involves identifying which metaphysical alternative is the actual one relevant to the causal sequence. This control involves aligning an actual speech action with the reasons that prompted it from the agent's perspective (Rainey, 2022, p. 513).

Endorsement and regulative control, as a way of identifying an actual causal sequence from intention to eventual speech output, are further complications in cases of synthetically realized speech. Owing to the ways in which devices will operate in a very practical sense, this suggests problems for speech ownership. The point essentially will be that skilled use of a speech device may require users to operate on reasons other than those attaching to a specific, desired utterance, e.g., if a device user knows that they need to exaggerate “p” sounds to have the device make optimal-sounding phonetic outputs, they might substitute speech actions for another in response to the specifics of the device's capabilities. Speaking optimally *through the device* might mean substitution among words, specific phrasings, etc. The set of reasons for a device user's particular speech production may come to include instrumental considerations regarding the limits of the device, over and above having reasons for saying *p*. While the speaker's meanings in an overall sense might remain pretty much intact, the rout to specific utterances could be bounded by non-linguistic reasons and considerations. Endorsement in those cases might well be constrained, and regulative control over actual causal sequences curtailed.

To explore this and highlight some questions about speech ownership in BCI contexts, first a general sketch of a speech system will be provided. This will help to gain a sense of the kinds of limits systems might introduce in virtue of their features. Next, some dimensions of the pragmatics of speech will be laid out. Having detailed this, the article will analyze how these points about BCI speech system design and the pragmatics of speech might combine in a way that might impact on how BCI speech system users could relate to their reasons for saying *p, q*, or *r*. It will be claimed that system designs and limits could cause users to draw upon reasons besides those they would draw upon were it not for the BCI system. This fact will provide a basis for saying that BCI speech systems create intrinsic gaps in reasons-responsiveness, meaning users of such systems may have less than desirable control over their devices. This will amount to saying such users may enjoy less ownership over their BCI mediated speech outputs than is desirable. Finally, as a consequence of this speech-ownership gap, it will be suggested that synthetic speech realized through devices should be considered not as continuous with standard “organic” speech, but as a novel communicative practice.

## 2. General sketch of a speech system

The idea of speech systems based on non-externalized verbal inputs has been around for a while. These have included as proposed inputs articulatory movements, vocal tract ultrasound signals, electroencephalogram (EEG), and signals from intracortical recordings of articulatory-motor areas in the brain, among others (Denby et al., 2010; Wee et al., 2010; Brumberg et al., 2011; Bocquelet et al., 2016; Martin et al., 2019). For the purposes of this investigation into reasons-responsiveness and speech ownership, no specific format for a speech system is required. Rather, the skilled use of an interface mediating speech from the speaker to the external world via technical means is all that is required.^1^ Whether a device user carefully imagines speaking, producing neural correlates of covert speech, or they produce non-vocalized articulator movements, or anything else, will count toward their skilled use of their device. How that skilled use relates to eventual synthetic speech outputs is what will be of interest here, again, agnostic over what exact format the synthetic speech takes (it could be speech externalized by a vocoder, text on a screen, morse code, or any conceivable communication medium). What this paper examines is the relationship between the reasons behind skilled use and how these might vary, or be impacted upon, by the very presence of an interface between speech intentions and a device used to technically realize some speech output.

**Figure 1:**
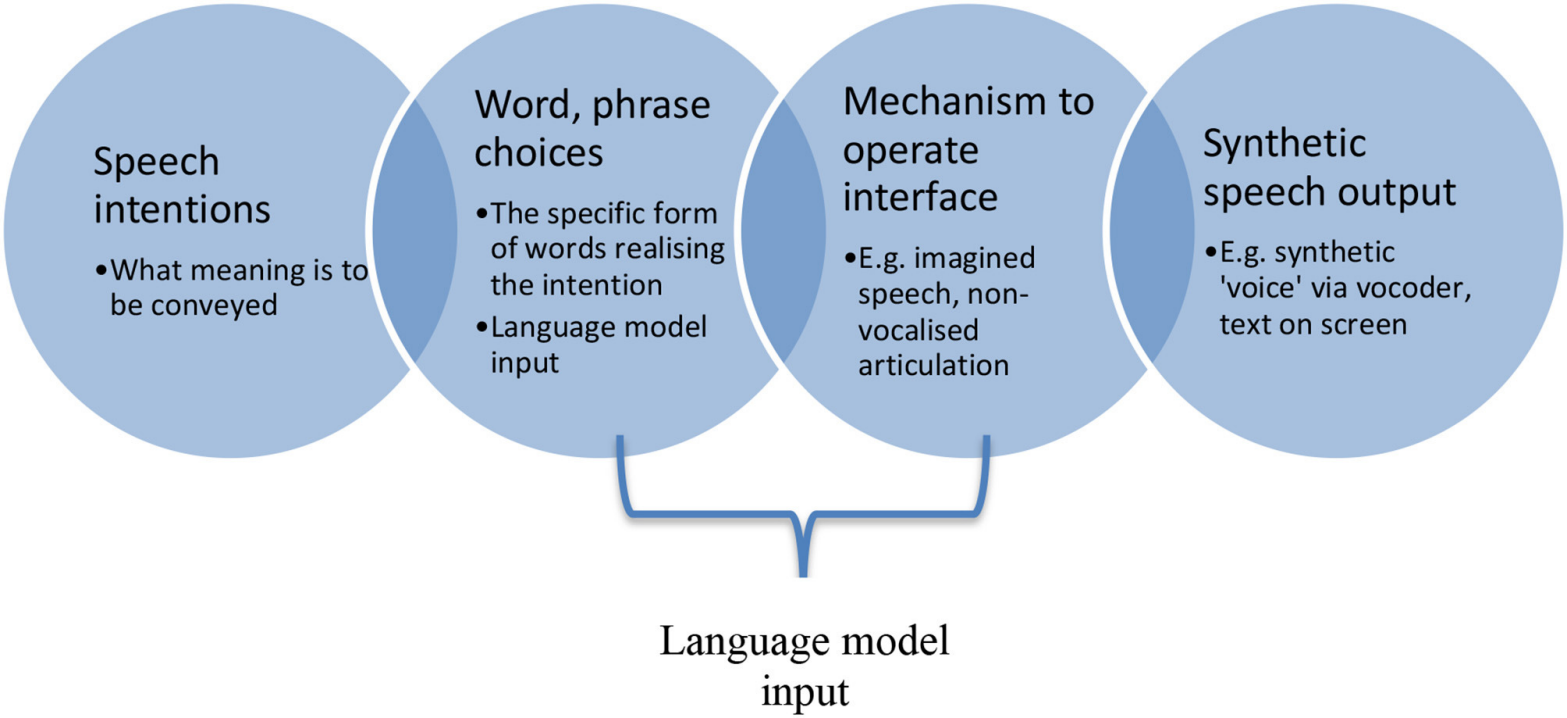
A very generic workflow for a speech-realizing interface system, from speech intention to synthetic speech-production by a device.

In Figure 1, between speech intention and word or phrase choice, a constraint will emerge based in how proficient the speaker is with respect to the language in which they intend to speak. A rich vocabulary enjoyed by a speaker of a first language will permit more possibilities than would the relatively impoverished grasp of a language picked up for, say, a specific context, or for communicating on a holiday. Between chosen verbal forms and eventual speech output, the exact mechanism of interfacing between intention and output will play a role. Some interfaces may be easier to pick up than others for different users, and each will produce different demands on the user of the device. For instance, imagined speech as a control mechanism for a synthetic speech device might require a user to internally “shout” a word or phrase so as to realize a neuroelectrical signal neuroanatomically equivalent enough to overt speech, such that the system can detect and decode it.

Whether a user finds this relatively easy or difficult, the interface nevertheless produces a burden and requires specific effort that, over time, can be improved, as might any skill. In order to maximize speed and efficiency, and to permit the system and the user to co-improve, a language model would ideally appear as part of the mechanism to operate the interface. A language model here would amount to an artificially intelligent processor of device-user inputs such that from word and phrase choices subsequent ones could be predicted in advance of their actual production. This predictive element would help to speed up the system overall, in priming the synthetic speech output in terms of likely onward speech, as well as reducing some burden on the device user in permitting them to prompt the device toward desired speech outputs without necessarily having to produce them all.

From the user-to-device side, the kind of skill required to operate a speech device on this template is relatively easy to understand—users can get used to the interface their device operates with and get better at using it. But there is, I suggest, the possibility of device-to-user constraints also relevant to the interface, and that can have effects upon word and phrase choice or affect speech intentions. To continue with the current example of imagined speech being “shouted” internally, for instance, this might be quite burdensome for longer bouts of speech.

The act of virtually shouting one's thoughts might be difficult to keep up with or even to occlude ongoing thought processes, proving ultimately distracting from the original speech intention. The specific format of synthetic device output too will include technical specifications that will for good or ill introduce constraints on the realizability of speech. For instance, a synthetic speech system might have particular difficulty in realizing sounds associated with consonants, in contrast with the easy production of vowel sounds. This “difficulty” might stem from specific software or hardware parameters, and amount practically to overlaps between Fs and Ss, or between Bs and Ps, and thereby ambiguity for listeners in the speech the device can produce. As such, the device itself might introduce extra interpretive burdens for audiences, meaning that they might have to take a moment longer than otherwise they might in order to get what is being said via a synthetic speech device. The language model too may prove a double-edged sword in perhaps being too intrusive, or too often mistaken, such that the device user becomes frustrated with repeated mis-prediction. This could, for example, result from an artificially intelligent language model that had been trained in a dialect other than the device user's own (e.g., American English vs. Hibernian English).

These observations about some of the technical constraints on speech production potentially latent in any BCI synthetic speech device should already be enough to suggest that skilled use of such devices might include deviation from intended speech in order to maximize the chances of best realizing speech outcomes. At any rate, this is a possibility that requires further examination in terms of pragmatics, and then in terms of the reasons that speakers have for saying what they say.

## 3. Pragmatics and speech

Pragmatics in general, from a philosophical point of view, includes the analysis of utterances as a fundamental unit of action. What competent speakers of a language do with their words is the unit of study in pragmatics, and it draws upon things like speaker intentions, linguistic conventions, semantic properties of words, grammatical rules in language, how contexts can be construed, and more. One very general way of looking at the variety of elements at work in pragmatically accounting for language is to think about *what is done with what is said*. One job is therefore to decide what is said. Among the elements of interest in terms of this, a distinction can be made between “near side pragmatics” and “far side pragmatics”. Asking “what is said” with an utterance can have at least two meanings, one concerning, e.g., word choices, another concerning the meaning of an utterance more widely construed—what is achieved by making that utterance. Near side pragmatics deals with the former, far side the latter. For example, the difference between the next two sentences is clear from a lexical and grammatical point of view, but perhaps less so as an utterance, owing to phonetic properties of speech:

1. Let us know what you think
2. Lettuce no watch youth ink
3. The cat sat on the mat
4. Thicket satin dim at
5. Put your money on the table
6. Putsch ermine eon debt Abe all

Sentences 1, 3, and 5 are straightforwardly understandable. Despite being comprised of real words in English, 2, 4, and 6 meanwhile, are literal nonsense as rendered on the page. For instance, sentence 1 is a straightforward request for an evaluation of some kind. Sentence 2 is literal nonsense, but that (in certain accents) maps well onto something phonetically equivalent to 1. Whether sentence 1 or 2 were uttered to an interlocutor, that interlocutor might well respond with an evaluation. This would be a likely interpretation of the sounds heard because, in general, interlocutors try to make sense to one another and utter well-formed sentences. Ditto the other even-numbered sentences. As utterances, in various instances, they might stand as decent homophones for their more straightforward, odd-numbered counterparts. In terms of Gricean pragmatics, this aptness for interpretation as meaningful would be respecting the cooperative principle viz, “Make your conversational contribution such as is required, at the stage at which it occurs, by the accepted purpose or direction of the talk exchange in which you are engaged” (Grice, 1993, p. 26). The sudden production of literal nonsense in a talk exchange would be out of step with the purpose or direction, and so ascribing meaningfulness would be a reasonable interpretation, all things considered, regarding context and how people usually communicate. This would be a matter of far side pragmatics, where the sounds in 1 are taken to indicate a request for an evaluation, in 2 for a description of the cat's location, and in 5 for an instruction on how to pay.

If asked to describe sentence 2 as heard, the interlocutor might write down sentence 1 as that would represent the lexical form of the uttered request usually expected in the language. Given the apparent request for an evaluation of some kind, sentence 1 would fit neatly in an overall explanation of the situation: If asked “why did the interlocutor write sentence 1?” the answer would believe that it represents a request for an evaluation. If they wrote down sentence 2, as a phonetic equivalent to the heard request, this might be considered a failure of near-side pragmatic analysis, as their suggestion that the speaker had actually uttered 2 would not be well supported. It would not as neatly explain the relation between the sentence and the request for an evaluation, thereby indicating a failure with respect to Grice's maxim of relation: “Be relevant” (Grice, 1993, p. 27). In terms of relevance, the hearer of the utterance would have better reason to think of the speaker as uttering 1 over 2. The initial speaker, moreover, would be most likely to choose sentence 1 as a means of requesting an evaluation from their interlocutor since that is a neat, grammatically well-formed, and lexically simple way of putting things. One thereby fits with Grice's “supermaxim” of manner, “Be perspicuous” in avoiding obscurity of expression (Loc. Cit.). The speaker could utter indeed, possibly, sentence 2, as phonetically equivalent, but at the risk of being misunderstood or uttering nonsense, depending on pronunciation. They might utter sentence 2 as a joke, or a playful way of speaking, and thereby changing the mood in which the request for an evaluation is conveyed. The risk would be in uttering literal nonsense but hoping that it sounded close enough to be taken for sentence 1. You might say, from a normative near side pragmatics point of view, that if the speaker who wishes to request an evaluation in this case ought to utter sentence 1 rather than 2 since sentence 1 encapsulates the desired request linguistically.

Grice and others are interested in these sorts of matters to the extent that they can be related to speaker intentions. Following Grice's account, let (i_n_) indicate numbered intentions. A speaker's utterance is intended (i_1_) to elicit a response in an audience and the speaker intends (i_2_) that the audience recognize her (i_1_) and she intends (i_3_) that this recognition by the audience of (i_1_) will feature in the set of reasons that would be given by the audience for the production of their response (assuming they respond) (Grice, 1993, p. 92).^2^ Synthetic speech of the sort here discussed can straightforwardly throw this account into contrast with the reasons one might have for utterances. An intention i_1_ to request an evaluation as a response is revealed by sentence 1, for instance. That this intention be recognized as is intended i_2_ through producing an utterance that can clearly be taken as encapsulating that i_1_. The speaker then intends i_3_ that their utterance prompts and explains any response. But these intentions would not apply to sentence 2, with 2 being literal nonsense. Spoken, there is little to tell them apart. Assuming the skilled user uses their speech device fully in the knowledge of its limits and so forth, they may very deliberately use sentence 2 as their device trigger. While the intentional scheme just laid out still applies in a sense, it does not apply to the utterances the user of the device is really using in operating their device. If they use sentence 2, for instance, they do not intend that the sentence “Lettuce no watch youth ink” be recognized as a request for evaluation or that it feature in the reasons an interlocutor might produce for their response. The speech intentions attach to the speech acts insofar as they can be predicted to have appropriate effects on the audience. The reasons for the specific utterance of 2 rather than 1 will attach to knowledge of device constraints and limits and how best to ensure specific phonetic outputs.

The reasons for the production of some speech over other speech thus arise in tension with speech intentions. If a synthetic voice were to utter sentence 1 or 2, we might easily take either to embody linguistically a request for an evaluation, but knowing it to be a synthetic voice we might hesitate to say any corresponding intention had been revealed thereby. A synthetic voice might be part of an automated customer service phone system, for example, and so merely generate pre-defined sentences based on system aims. These would be instrumental or strategic instances of language, exploiting far-side pragmatic properties of spoken language in order to promote effects in hearers.

In terms of near side pragmatics, in the case of a synthetic voice, an engineer at some point in the system development would likely have input sentence 1 and tested the synthetic voice's rendering of it. In the event that it sounded somehow inadequate, the engineer might have ended up inputting sentence 2 since, for reasons of that system's sound generating properties, it made for a better rendering of the request for an evaluation. In this instance, an interlocutor might once again write down sentence 1 if asked to describe what the system “said”. They would be wrong in terms of near-side pragmatics, since it actually said sentence 2, but from a far-side perspective, the request for an evaluation would still be the meaning of the utterance, it would remain as what was being done with that utterance, and would reasonably be taken for it in terms of the cooperative principle, maxim of relation, and supermaxim of manner.

In this latter case of the engineer's decision making about phonetic adequacy of a synthetic voice, what's more important than speaker intention is the nature of spoken language and the structures of uttered sentences. While the synthetic voice might utter literal nonsense, if it has a better effect on the hearer owing to its phonetic properties, it provides better cues for the interlocutor to discern the reasons for that utterance than a lexically and grammatically correct sentence uttered in an unclear way. These variations relate to the form of the uttered sentence, but its content too might vary while retaining “the same” meaning to a reasonable extent. Consider:

7. Your feedback is welcome

Sentences 1 and 7 share a meaning to a reasonable extent, differing mainly in tone. Three is somewhat more impersonal, perhaps, which might be either more or less appropriate in some given circumstances. One could paraphrase 1 as 7 or vice versa without impacting very much on an understanding of the situation of the request for an evaluation. The retention of the semantic content of a sentence with a change in tone might be most significant where contextual elements are of special importance, such as in legal proceedings, medical decision-making, or other such situations. A too-chatty remark in a courtroom may affect proceedings by suggesting contempt for the seriousness of matters, for example. By contrast, an overly formal verbal approach to something like birthday greetings might strike an interlocutor as strange but produce little effect beyond puzzlement.

Form can differ, while retaining meaning. Content can be retained through verbal shifts. Near-side pragmatic choices can reflect contextual considerations through responding to circumstances by varying tone. Far-side pragmatic considerations can permit interlocutors or audiences to understand what's being done with a sentence uttered, despite changes in form, or despite being literal nonsense. These will be seen in what follows to form important dimensions in appreciating synthetic speech produced through systems like those operated in the basis of BCIs. How, overall, reasons and speech will come to be related will be considered and analyzed next.

## 4. BCI speech system users and reasons

If a BCI speech device user finds themselves choosing words according to standards influenced by known dimensions of the system, are they using near or far side considerations? Are they choosing the words and phrases primarily to match their speech intentions or primarily to maximize the intended effects of their utterances on their audience? And consider the system itself: what are its classifications and predictions best seen as modeling—near side concentrating on classifying word choices or far side anticipating communicative choices via language model?

Might there emerge a risk of a “pragmatic circle” (Korta and Perry, 2008), wherein words predominantly end up being chosen for the effect that they will have on the audience, and pragmatics becomes the primary mode for explaining speech action? A user of a speech device has access to only the lexicon built-in to the language model, and the forms of speech included in the same model. This might be comprehensive but could be at variance with the speakers' “organic” language competence. As such, in this sense, users of speech devices are using the device to communicate; they are not “speaking” in any usual sense of that word but operating on reasons geared toward the effective use of an inherently constrained communication device. How can we relate this to a synthetic speech production system like one operated via BCI? First, recall the near side/far side distinction in pragmatics:

- Near side: word choice includes user intentions through skilled use of the device and, e.g., imagined and covert speech. Also includes a language model which artificially intelligently classifies brain activity with respect to intended words, predicting words, next words, or whole sentences, based on a corpus of language on which it is trained.
- Far side: what's being done with the utterance, as judged by an audience who may be trying to divine speaker intentions, or more generally to locate the utterance of the specific spoken sentence in a linguistic and wider social context.

The general distinction here is between near side and far side as apparently dividing between speaker and audience. Speakers appear more closely involved with near side pragmatics, while audiences have mainly to deal with far side pragmatics. But in the case of a synthetic speech production device these can come to intersect or overlap as the user of the device must consider not only their speech intentions, but also practical constraints imposed by the device in terms of at least language model parameters and software and hardware constraints. For example, suppose the user knows the system is not great at dealing with sibilance, and produces unpleasant “s” sounds. They want to say: “That's some seriously strong espresso” but settle instead for “What a powerful little coffee.” The user might explicitly consider the effects the words will have in order to find equivalents for the phrase they would rather utter but for the limits of the device. Now the reasons for the utterance include considerations about the machine and how it works, besides what is intended to be said. This might indicate aesthetic conditions of ownership over utterances akin to those exemplified in oratory or poetry, but it can also be seen as a departure from widespread practices of ordinary speech.

What's more, the role of AI and a language model introduces an element of the system's intervention which has a bearing on both near and far side considerations: word choice as a matter of classifying brain activity like covert speech as corresponding to one word or another, and the language model as contextualizing predicted speech as for one purpose or another. The model might be able to act on very sparse input and generate concrete outputs nonetheless. For example, taking a case in which vowels are more easily decided than consonants in a cover speech paradigm, the following set of signals might emerge, where vowels correspond to letters and noise, and ambiguous signals are represented by “_”:

8. __e _a_ _a_ o_ __e _a_

This might feasibly be rendered as “the cat sat on the mat” based on a ChatGPT-like prompt as articulatory-motor neuron signal. The specific array of vowels in 8, with the specific pattern of inferred cadence, might straightforwardly (in terms of the data processing of the chat system) correspond to the whole sentence in a way it would not to a human interlocutor. An alternative sentence with the same vowel structure could be “the hat was on the cat” but this would, in terms of data and prior prevalence, be far less likely than “the cat sat on the mat”. A skilled synthetic speech device user could find themselves operating on the basis of reasons that have *prima facie* tensions with their speech intentions (i_1_) to (i_3_) when they take into account such factors as the input of a language model, besides other limitations. Intending to have speech input recognized as a request for feedback but having reasons to trigger a device through imagining literal nonsense do not coalesce except for the case of the synthetic speech device. Likewise, a system that predicts likely next words or sentences might be easier to work with rather than against. This might be the case despite a system's language prediction model being out of step with one's dialect or idiom. For a better effect on an audience, the device user might have good reasons for acquiescing in the predictions of the model despite departure from their own specific speech habits.

In terms of reasons for speaking one way rather than another, at least three dimensions are relevant. As highlighted in Mecacci and Santoni de Sio's (2020) discussion of dual mode vehicles, *operational, tactical*, and *strategic* reasons play a role in action. Furthermore, these can relate in specific ways to types of intention more or less proximal to the action performed. Mecacci and Santoni di Sio are explicitly engaged in a project of expanding conventional philosophy of action to include reasons-responsiveness over not just action, but also systems:

“Whereas in the traditional philosophy of action, practical reasoning was meant as an explanatory aid to make sense of the relationship between human reasons to act and human action, we propose to use the structure of practical reasoning to make sense of the relationship between human reasons and *the behaviour of systems which include human and nonhuman agents*” (Mecacci and Santoni de Sio, 2020, p. 108).

Mecacci and Santoni di Sio's reference to operational, tactical, and strategic reasons reflecting facets of control pertain directly to driving. If we consider driving a car as an example of action, steering (operational) would be done for the reason of overtaking (tactical), which itself is done for the reason of going home (strategic). But overtaking in a specific place is done for the reason of its being legal and safe according to wider considerations of respect for values and norms. These are in turn apt to be mapped onto different types of intentions, at increasing “distances” from the specific actions undertaken. Immediate proximity to action, steering, is on a spectrum with distal intentions, going home (tactical reasons we can assume to be more or less proximal or distal, depending on what's happening). This makes room for intentions being relevant both in terms of considering specific actions, as well as for coordinating action more widely (cf. Bratman, 1984). This model of reasons and intentions for meaningful human control over systems, in relating to philosophy of action *per se*, is amenable to translation into other domains where such control is important. In the case of a synthetic speech device, it is particularly useful, and enables us to relate the foregoing analysis in terms of pragmatics and speech to reasons and ownership over that speech. Here, specific covert speech (operational) might be chosen for the reason of device constraints (tactical) for the reason of being clearly understood concerning a wish, desire, or expression (strategic). All of that might fall under the more general reason of respecting constraints in the device, including hardware and software limits. Overall, such reasoning too will be bound by norms and values present in language, such as Grice's cooperative principle.

Mastery of the device, insofar as one makes oneself understood successfully, might require mastery of the language (e.g., knowing lots of synonyms for substituting phrases) but this could diverge from ownership conditions for speech because one's reasons for speaking respond not only to speech desires and intentions, but also to instrumental considerations regarding the machine. Proximal, specific intentions in moment-to-moment operation of the device via covert speech may reflect distal, coordinative, intentions relating to either sheer control of the device or in very generally making oneself understood. Here, the kinds of “proximity” of intentions mirror the near/far side distinction in pragmatic terms—near-side pragmatics and proximal intentions regarding word choices map onto one another, while far-side pragmatics and distal intentions regarding making oneself understood map onto one another.

**Figure 2:**
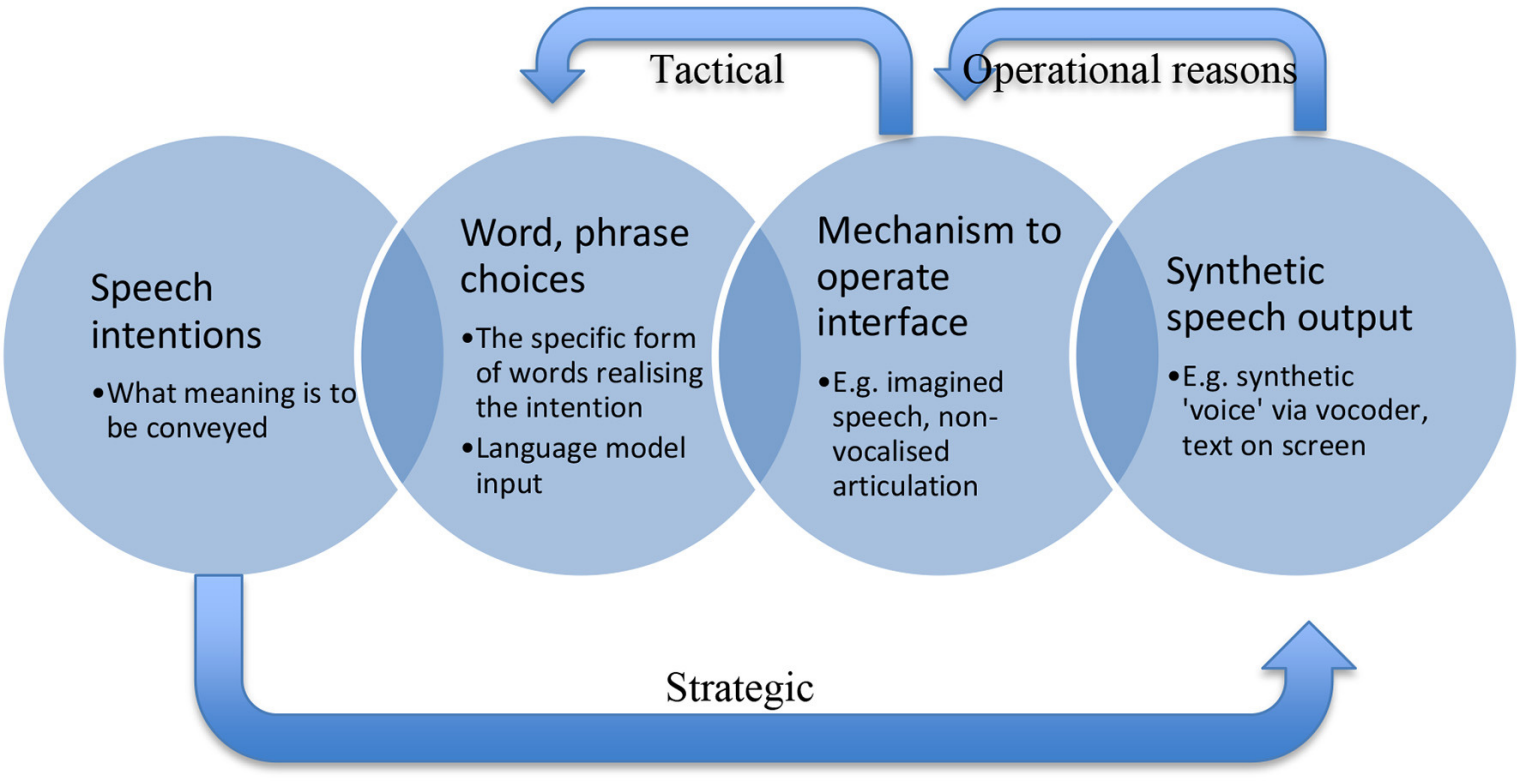
A modified schema from Figure 1, omitting input of the language model but including types of reasons and their influence on synthetic speech production. Strategic reasons regarding making oneself understood provide an overall reason to aim for a specific speech output, respecting such factors as Grice's cooperative principle, but skillful use of a device will include known constraints of the hardware and software. Skilled use thus provides tactical reasons for choosing specific word forms and operational reasons to trigger the device in specific ways given specific constraints.

In Figure 2, a complex picture is shown whereby a speech device user will have good reasons aside from linguistic reasons to choose specific words and phrases, and to engage with their speech device in specific ways. This scheme of device operation could be at variance with the speakers' “organic” language competence. As such, in this sense, users of speech devices are using the device to communicate, rather than “speaking” through it, in an appreciably usual sense of that word. Tactical reasons are analogous with the plan to steer a car, with operational reasons motivating the actual pulling of the wheel one way or another in its service. Here, “steering” is the overall word or phrase choice aligning with tactical reasoning, while operational reasons for specific “pulls of the wheel” are aligned with triggering the device in specific ways like imagining spoken syllables, words, or sentences. Overall, the strategic reasons are provided by trying to make oneself understood—the level of speech action rather than preoccupation with a specific utterance.

The skilled user will have non-linguistic reasons for using their device to output specific utterances. They may have learned over time that the system responds better to imagining the literal nonsense “Lettuce no watch youth ink” rather than the correct “Let us know what you think,” for instance. That skilled user would therefore “speak” literal nonsense in the service of making themselves understood, based in their skillful deployment of their linguistic device. Ditto for remarks make about “powerful little coffees” when it was known to the device user that the sibilance of “espresso” might trip the device up through fuzzy or distorted sound reproduction, trouble in reproducing certain consonants, or other such practical limits. Skilled use, this suggests, includes a device user's ability to think of what it is they wish to be understood to be saying, and then drawing upon an array of operational and tactical reasons in order to make their device produce outputs in the service of their overall strategic reason. This has ramifications for ownership of the speech produced via a BCI speech device.

## 5. Control and ownership revisited

Guidance control was that sort of control exercised over a device in order to make it produce desired outputs. In this case, those are specific synthetic verbal outputs. This is a causal power over the device. Yet in terms of what has just been examined, guidance control over a speech device put to use by a skilled user will include responsiveness to constraints inherent to the system, such as might emerge from the input of a language model, or from software or hardware properties. This means that if a device user is a skilled user, they will exercise guidance control over their device in light of an array of factors not limited to the linguistic. Specifically in terms of Grice, the user of the device might, in the service of the cooperative principle, have to violate relevance and manner through uttering literal nonsense (e.g., “lettuce no…”), or being circumlocutory (e.g., “powerful little coffee”). These synthetic speech outputs would be the best means available for having the desired communicative effects on the audience. But their genesis would be constrained by linguistic and extra-linguistic factors. Primarily, the overarching reason a speech device user will have in trying to produce one utterance rather than another will be strategic, and aimed at the level of speech action, with speech production in an instrumental relation to it.

Ownership over speech was already related to endorsement after the fact, owing to the ways in which semantically accurate speech derived from a speech intention could nevertheless be open to interpretation in a way the speaker disavowed. Meanwhile, relating utterances to an actual causal pathway, and thereby exercising regulative control over them in a specific sense, highlighted another dimension of control such that ownership could be understood. But in the sorts of cases arising here, endorsement may only go so far. A device user might endorse their utterance to the extent that it was the best means of trying to realize a communicative effect, within the bounds of the cooperative principle. But the reasons they might have had to produce the specific triggers for their device might include tactical and operational reasons based in familiarity with overall system constraints (e.g., distorted sibilance or regional affect in the AI language model). In this way, the actual causal sequence leading to a specific output might well depart from anything a speaker would wish to say, all other things being equal. It may, as with “lettuce no…”, amount to gibberish.

A skilled user may have a good grasp of the limits and strengths of their speech device and so deploy a range of phrases and word choices so as to downplay limits and boost strengths. This might mean that their speech intentions are realized at the level of speech action—the message gets across—but the intended phrasing is not the exact one the speaker would have preferred to use, all things considered. They might have tactical reasons that condition their speech intentions given knowledge of system limits and words of phrases most easily reproduced by the system. The skilled user might have operational reasons for specifically triggering the system in a given way, again owing to knowledge of practical and technical limits. This might amount to producing imagined speech not of the desired sentence, but of a phonetic equivalent that is literally nonsense but that will produce the best effect.

## 6. Conclusions

In terms of endorsement and regulative control, there are potentially significant departures from standard speech expectations when it comes to a synthetic speech device. These foreshorten the extent or the quality of ownership over speech that a device user might enjoy. Endorsement might be expected only so far, as skillful use will circumscribe a narrower than otherwise expected speech repertoire. Meanwhile, regulative control will be exercised in ways that include awareness of deploying literal nonsense in the service of producing strategically appropriate sounds. This chance of deformed ownership is—almost paradoxically—based in the fact of the user's skill with the device. Guidance control will be exhibited through the skillful use of the device in reliably producing intended outcomes. But that reliability comes with the unusual realization that nonsense might be in the service of sense. A *strategy* of “getting the best” out of a speech device might amount to producing the *tactically best* utterances, not quite what would ideally be endorsed, through sometimes *atypical operational* means like imagining literal nonsense. Speech intentions, in short, and reasons for specific speech behaviors come apart.

While, in the grand scheme of things, these somewhat abstract considerations might not be taken to much affect what can and cannot be done with synthetic speech systems, they are nonetheless important to consider. In cases of therapeutic uses of speech systems, it is perhaps least likely that these considerations would lead to a substantial critique of synthetic speech. If it is technically possible to restore a communicative capacity to someone for whom that capacity has been damaged, the more the better. Even in this case, though, the reflections here on speech ownership and the description of atypical speech production suggest an increased interpretive latitude for audiences. Enhanced interpretive charity for those communicating in line with their speech intentions but also with device-derived strategic, operational, and tactical reasons playing a role, appears justified. For uses beyond the therapeutic, including emerging potential use cases like in military or human enhancement contexts, these reflections ought to substantiate the realization that, despite superficial and functional similarities, synthetic speech systems and “organic” speech might depart from one another in quite significant ways. Synthetic speech systems may be best thought of as tools implementing novel communicative practices modeled on the familiar, not as technically mediated continuations of the familiar.

## Data availability statement

The original contributions presented in the study are included in the article/supplementary material, further inquiries can be directed to the corresponding author.

## Author contributions

SR fully conceptualizing, drafting, and editing this article.

## Footnotes

^1^The account developed here is counter to the treatment of “skilled use” developed in Drosselmeier and Sellmaier (2021) for whom skilled use can be considered a “basic action”, i.e., an action done not by doing another action. Pursuit of this angle in this context could be of genuine interest but is for now out of scope.

^2^There are great reasons not to run with an account of linguistic meaning and communication that is overly concerned with intentions, but they are out of scope here. See Heck (2006) for a direct way out from Grice's specific intentionalist preoccupation.
